# Cost-effectiveness and resource use of implementing MRI-guided NACT in ER-positive/HER2-negative breast cancers in The Netherlands

**DOI:** 10.1186/s12885-016-2653-y

**Published:** 2016-09-05

**Authors:** Anna Miquel-Cases, Lotte M. G. Steuten, Lisanne S. Rigter, Wim H. van Harten

**Affiliations:** 1 Department of Psychosocial Research and Epidemiology, Netherlands Cancer Institute, Plesmanlaan 121, Amsterdam, 1066 CX The Netherlands; 2 Hutchinson Institute for Cancer Outcomes Research, Fred Hutchinson Cancer Research Center, 1100 Fairview Ave. N., P.O. Box 19024, Seattle, USA; 3 Department of Medical Oncology, The Netherlands Cancer Institute, Plesmanlaan 121, Amsterdam, 1066 CX The Netherlands; 4 Department of Healthcare Technology and Services Research, University of Twente, Drienerlolaan 5, 7522 NB Enschede, The Netherlands

**Keywords:** Cost-effectiveness, Resource utilization, Breast cancer, Neoadjuvant chemotherapy, Response monitoring, MRI

## Abstract

**Background:**

Response-guided neoadjuvant chemotherapy (RG-NACT) with magnetic resonance imaging (MRI) is effective in treating oestrogen receptor positive/human epidermal growth factor receptor-2 negative (ER-positive/HER2-negative) breast cancer. We estimated the expected cost-effectiveness and resources required for its implementation compared to conventional-NACT.

**Methods:**

A Markov model compared costs, quality-adjusted-life-years (QALYs) and costs/QALY of RG-NACT vs. conventional-NACT, from a hospital perspective over a 5-year time horizon. Health services required for and health outcomes of implementation were estimated via resource modelling analysis, considering a current (4 %) and a full (100 %) implementation scenario.

**Results:**

RG-NACT was expected to be more effective and less costly than conventional NACT in both implementation scenarios, with 94 % (current) and 95 % (full) certainty, at a willingness to pay threshold of €20.000/QALY. Fully implementing RG-NACT in the Dutch target population of 6306 patients requires additional 5335 MRI examinations and an (absolute) increase in the number of MRI technologists, by 3.6 fte (full-time equivalent), and of breast radiologists, by 0.4 fte. On the other hand, it prevents 9 additional relapses, 143 cancer deaths, 23 congestive heart failure events and 2 myelodysplastic syndrome/acute myeloid leukaemia events.

**Conclusion:**

Considering cost-effectiveness, RG-NACT is expected to dominate conventional-NACT. While personnel capacity is likely to be sufficient for a full implementation scenario, MRI utilization needs to be intensified.

## Background

Neoadjuvant (preoperative) chemotherapy (NACT) is equally effective as adjuvant chemotherapy in breast cancer [1], while offering the possibility of tailoring therapy based on tumour response at monitoring [2]. Among non-invasive imaging modalities for response monitoring, contrast-enhanced magnetic resonance imaging (MRI) is generally regarded as the most accurate for invasive breast cancer. It has good correlation with pathologic complete response (pCR), the most reliable surrogate endpoint of survival [3–5].

Researchers in the Netherlands Cancer Institute (NKI) have previously published criteria for monitoring NACT response with MRI [6]. The research confirmed its prediction for pCR in the triple negative breast cancer subtype [7], but not in oestrogen receptor-positive (ER+) and epidermal growth factor receptor 2- negative (HER2-) tumours. This was not an unexpected finding, given the known low rates of pCR in ER-positive/HER2-negative tumors [8, 9] make it an unsuitable measure of tumour response in these tumours. Hence, to investigate their benefit from response-guided NACT (RG-NACT), a subsequent study from this group used serial MRI response monitoring as a readout of response [10]. In this study, unresponsive tumours to the first chemotherapy regimen were switched to a second, presumably, ‘non-cross-resistant’ regimen. Upon study completion, the tumour size reduction caused by the non-cross-resistant regimen was similar to that in initially responding tumours after the first regimen. Furthermore, relapse frequency in both groups was similar. These observations suggested that ER-positive/HER2-negative tumours do benefit from RG-NACT with MRI, despite not reaching pCR. These results are in line with those from the German Breast Group [11], which also showed survival advantage from RG-NACT in ER+ patients.

Compared to traditional NACT, RG-NACT has thus shown to positively influence ER-positive/HER2-negative patients’ survival, yet comes at additional monitoring costs. Its onset costs may however be offset by a reduction in the subsequent medical costs. This can be explored via probabilistic cost-effectiveness analysis (CEA), which quantifies the probability and extent to which RG-NACT is expected to be cost-effective compared to conventional NACT as based on current evidence. Such information is of interest for health-care regulators who, under the pressure of limited resources, are increasingly using cost-effectiveness as a criterion in decision-making [12].

An important goal for decision-makers is the implementation of cost-effective health-care interventions into routine clinical practice. Yet this can often be jeopardized by the lack of attention given to resource demands [13]. Implementation as described in a CEA may not always be feasible, as this assumes that all physical resources (i.e., doctors, scanners, drugs) required by the new strategy are immediately available, regardless of actual supply constraints (or likely demand). Ignoring these constraints may result in negative consequences, from low levels of implementation through to the technology not being implemented at all [13]. Resource modelling is a method that quantitatively captures the resource implications of implementing a new technology. While this approach has scarcely been used in health-care decision-making, it can be of great help to health services planners who are challenged by implementation issues normally not addressed in CEAs.

Our aim is thus to estimate the expected cost-effectiveness and resource requirements of implementing RG-NACT with MRI for the treatment of ER-positive/HER2-negative breast cancers using The Netherlands as a case study population.

## Methods

This study followed the Consolidated Health Economic Evaluation Reporting Standards (CHEERS) checklist and did not require ethical approval [14].

### Treatment strategies

**Fig. 1:**
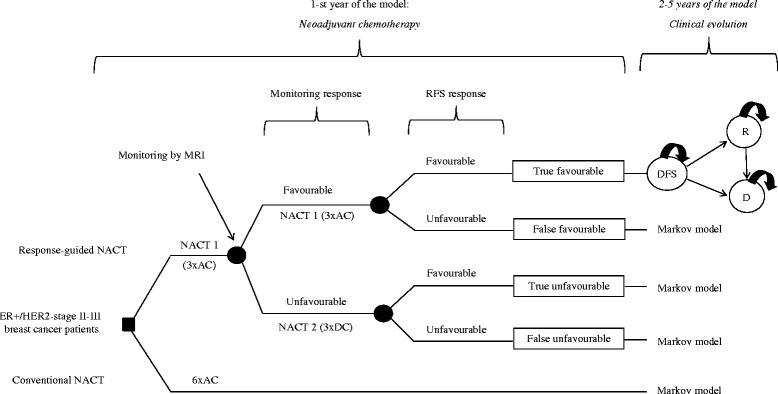
Decision analytic model to compare the health-economic outcomes of treating ER-positive/HER2-negative stage II-III breast cancer patients with response-guided NACT vs. conventional-NACT. Decision nodes (■); patient or health provider makes a choice. Chance nodes (●); more than one event is possible but is not decided by neither the patient or health provider. Abbreviations: NACT = neoadjuvant chemotherapy; RFS = relapse free survival; DFS = disease free survival; R = relapse; D = death; AC = cyclophosphamide, doxorubicine; DC = docetaxel, capecitabine

Two strategies were considered for the treatment of ER-positive/HER2-negative breast cancer women; RG-NACT and conventional-NACT (Fig. 1). RG-NACT followed our single-institution neoadjuvant chemotherapy program [10]: treatment with NACT 1 (AC, doxorubicin 60 mg m − 2 and cyclophosphamide 600 mg m − 2 on day 1, every 14 days, with PEG-filgrastim on day 2) for three courses (3x) followed by MRI scanning and subsequent classification into ‘favourable’ or ‘unfavourable’ responders to NACT defined by previously published criteria [6]. In short, reduction of more than 25 % in the largest diameter of the tumour at late enhancement on the interim MRI relative to the baseline MRI was regarded as a ‘favourable’ response. All other responses were classified as ‘unfavourable’. Favourable patients continue with additional 3×NACT 1, and unfavourable patients switch to 3×NACT 2 (DC, docetaxel 75 mg m − 2 on day 1, every 21 days and capecitabine 2 × 1000 mg m − 2 on days 1–14). Conventional-NACT represented current practice: treatment with 6×AC. Following NACT, all patients underwent surgery, radiation therapy when indicated and at least 5-years of endocrine treatment according to protocol.

### Implementation scenarios

We performed the cost-effectiveness and resource modelling analysis for two implementation scenarios in the Netherlands, i.e. current implementation and full implementation. These scenarios were adopted in a hypothetical cohort of 6306 patients, reflecting the Dutch target population of stage II/III ER-positive/HER2-negative breast cancers. These are patients with the same baseline characteristics as those of our neoadjuvant chemotherapy program, and thus, where RG-NACT seems beneficial [10]. The current implementation scenario is defined as the number of stage II/III ER-positive/HER2-negative breast cancer patients currently treated with RG-NACT divided by all stage II/III ER-positive/HER2-negative breast cancer patients. The full implementation scenario considers the use of RG-NACT in the entire stage II/III ER-positive/HER2-negative breast cancer population. Although this is not entirely likely, there is always a percentage of non-compliant providers, we decided to present the maximum possible resource use of RG-NACT. The number of patients currently treated with RG-NACT was calculated as the number of scans performed in the Netherlands (assuming 1 scan/patient) [15] minus the number of scans performed for other disease areas than oncology [16], other cancers than breast [17], other applications than guiding response to therapy [18], other stages than II/III [19], and other receptor expressions than ER-positive/HER2-negative [20]. The entire stage II/III ER-positive/HER2-negative breast cancer population was estimated by multiplying the 2013 breast cancer incidence in the Netherlands (The Netherlands Cancer Registry) by the proportion of patients with stage II/III ER-positive/HER2-negative breast cancer (calculations presented in Table 1).

**Table 1:**
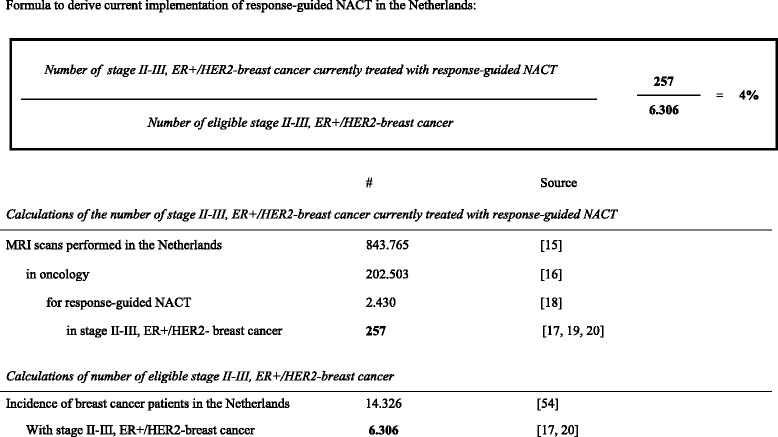
Current implementation scenario calculation [15–20, 54]

### Model overview

We developed a Markov model to estimate mean differences in clinical effects and costs of treatment with RG-NACT vs. conventional-NACT from a Dutch hospital perspective. For each treatment strategy, the model simulated the transitions of a hypothetical cohort of stage II/III ER-positive/HER2-negative breast cancer patients of 50 years old over three health-states: disease free (DFS), relapse (R, including local, regional and distant) and death (D, including breast cancer and non-breast cancer), during a 5-year time horizon (Fig. 1). The model was programmed in Microsoft Excel (Redmond, Washington: Microsoft, 2007. Computer Software).

Upon completion of the NACT intervention, patients in each cohort entered the model in the DFS state (Fig. 1). Patients treated under the RG-NACT strategy entered the DFS model state classified as true-favourable, true-unfavourable, false-favourable and false-unfavourable respondents of NACT at monitoring by using the 5-year RFS (relapse free survival) as the “gold standard” for NACT response. This was considered a sensible assumption to capture all relapses related to NACT response [21]. Definitions for true-favourable, true-unfavourable, false-favourable and false-unfavourable respondents are presented in Table 2.

**Table 2:** Definitions of true-favourable, false-favourable, true-unfavourable and false-unfavourable used in our study

Group of patients	Definition
True favourable	Patient that is classified as favourable at monitoring (criteria [7]), continues receiving NACT 1, and after 5 years of follow up is classified as favourable due to absence of relapse event
False favourable	Patient that is classified as favourable at monitoring (criteria [7]), continues receiving NACT 1, and after 5 years of follow up is classified as unfavourable due to presence of relapse event
True unfavourable	Patient that is unfavourable at monitoring (criteria [7]), switches to NACT 2, and after 5 years of follow up is classified as favourable due to absence of relapse event (*the underlying assumption is that the patient was not responding to NACT1 but did to NACT 2, thereby demonstrating that monitoring classified the patient properly)*
False unfavourable	Patient that is unfavourable at monitoring (criteria [7]), switches to NACT 2, and after 5 years of follow up is classified as unfavourable due to presence of relapse event (*the underlying assumption is that the patient was responding to NACT1 and did not to NACT 2, thereby demonstrating that monitoring classified the patient wrongly)* ^a^

^a^Although we are aware that in the ‘False favourable’ group there could be patients irresponsive to both NACT 1 and 2, as the design of the RG-NACT does not allow distinguishing them, we had to make such an assumption

In year 1 of the DFS health-state, patients were attributed the costs and health related quality-of-life (HRQoL) of the NACT intervention, except when there was an incidental MRI finding or when they suffered from chemotherapy-related toxicities (Terminology for Adverse Events grades 3 and 4 [22]); vomiting, neutropenia, hand-foot-syndrome (HFS), desquamation and congestive heart failure (CHF) [23, 24]). In these situations, there was NACT interruption and temporary changes in costs and HRQoL, except for HFS and desquamation. For these toxicities there is no other curative treatment than time, thereby, they were exempt of costs. From the DFS health-state, patients could either move to the R health-state, i.e., ‘relapse event’; move to the D health-state, i.e., ‘non-breast cancer death event’; or stay in the DFS health-state, i.e., ‘no event’. From the R health-state, patients could either move to the D health-state, i.e., ‘breast cancer or non-breast cancer related death event’; or stay in the R health-state, i.e., ‘cured relapse’. We assumed that patients could only develop one relapse. In the 5th-year of the model, patients could incur long-term NACT-related toxicities, including myelodysplastic syndrome (MDS) and acute myeloid leukaemia (AML) [25].

### Model input parameters

Input model parameters are presented in Table 3.

**Table 3:** Input model parameters

Parameter			mean	SE	Parameters^a^	Distribution		Source
Clinical data								
Monitoring performance^b^ (proportions)								
True favourable			0,53	0,04	0,53/0,04	Dirichlet		[10]
True unfavourable			0,24	0,05	0,24/0,05	Dirichlet		[10]
False favourable			0,17	0,07	0,17/0,07	Dirichlet		[10]
False unfavourable			0,07	0,09	0,07/0,09	Dirichlet		[10]
Chemotherapy related toxicities								
Vomiting		3×AC	0,05	0,02	5/98	beta		[24]
		3×DC	0,24	0,04	24/77	beta		[24]
HFS		3×DC	0,22	0,04	23/80	beta		[24]
Neutropenia		3×AC	0,85	0,04	86/15	beta		[24]
		3×DC	0,72	0,04	74/29	beta		[24]
Desquamation		3×DC	0,05	0,02	5/98	beta		[24]
CHF		3×AC	0,002	0,20	1/359	beta		[23]
		6×AC	0,02	0,60	11/349	beta		[23]
AML/MDS		3×AC	0,003	0,001	12/4471	beta		[25]
		6×AC	0,005	0,001	12/2372	beta		[25]
Transition probabilities								
Relapse								
RG-NACT; False favourable/unfavourable		Tp1	0,14	0,06	4/24	beta		[10]
		Tp2	0,29	0,08	8/20	beta		[10]
		Tp3	0,47	0,09	13/15	beta		[10]
		Tp4	0,44	0,09	12/16	beta		[10]
		Tp5	0,40	0,09	11/17	beta		[10]
RG-NACT; True favourable/unfavourable		Tp1^2^-5	0,00	NA	-	fixed		assumption
HR RFS (RG-NACT vs. conventional-NACT)			0,50	0,20	0,50/0,20	Normal truncated		assumption
Conventional-NACT		Tp1	0,03	-	-	-		[10]
		Tp2	0,06	-	-	-		[10]
		Tp3	0,08	-	-	-		[10]
		Tp4	0,05	-	-	-		[10]
		Tp5	0,04	-	-	-		[10]
Breast cancer specific death								
False favourable/unfavourable		Tp1	0,00	NA	-	fixed		assumption
		Tp2	0,04	0,02	5/109	beta		[27]
		Tp3	0,12	0,03	14/100	beta		[27]
		Tp4	0,06	0,02	7/107	beta		[27]
		Tp5	0,19	0,04	22/92	beta		[27]
HR BCSS (RG-NACT vs. conventional-NACT)			0,64	0,13	0,64/0,13	normal		[11]
Conventional-NACT		Tp1	0,00	NA	-	fixed		assumption
		Tp2	0,06	-	-	-		[27]
		Tp3	0,19	-	-	-		[27]
		Tp4	0,09	-	-	-		[27]
		Tp5	0,28	-	-	-		[27]
Utilities								
Chemotherapy			0,62	0,04	94/58	beta		[39]
Neutropenia			0,53	0,01	557/488	beta		[40]
Anxiety			0,68	0,06	40/19	beta		[43]
Vomiting			0,52	0,08	17/16	beta		[41]
HFS			0,50	0,10	12/12	beta		[41]
Desquamation			0,59	0,01	1041/721	beta		[40]
CHF (average grade III/IV)			0,55	-	-	beta		[42]
CHF grade III			0,59	0,02	360/250	beta		[42]
CHF grade IV			0,51	0,05	52/50	beta		[42]
MDS/MLA			0,26	0,01	500/1423	beta		[55]
DFS			0,80	0,03	196/49	beta		[39]
R (average loco-regional and metastatic)			0,73	-	-	beta		[39]
Loco-regional relapse			0,68	0,03	226/104	beta		[39]
Metastatic relapse			0,78	0,04	104/30	beta		[39]
Scenarios and resource modelling								
Incidental findings								
All			0,18	0,01	270/1265	beta		[29]
Malign			0,20	0,02	55/270	beta		[29]
MRI contraindications								
Impaired renal function			0.07	0.1^c^	0.45/5.54	beta		[49]
Gadolinium allergy			0.0003	0.01^d^	0.08/29	-		[44]
Body ferrous parts			0.58	0.1	0.26/4.21	beta		[45]
Claustrophobia			0.02	0.1	0.02/0.94	beta		[48]
Uptake			0.04		20-100 %	fixed		assumption
MRI technologists with ATS			0.26		-	fixed		[50]
Costs								
Parameter		Unit costs	Unit measure	Mean resource use	Mean cost	SE^e^	Distribution	Source
Chemotherapy								
6×AC	Doxorubicin	€204	90 mg	5,3	€1306	€326	Gamma	[31]
	Cyclophosphamide	€45	1080 mg	6,4	€239	€60	Gamma	[31]
	Peg-filgrastim	€849	1 mg	6	€5096	€1274	Gamma	[56]
	Pharmacy preparation	€45	Per course	6	€267	67	Gamma	NKI
	Day care	€286	Day	6	€1718	€430	Gamma	[30]
	Oncologist’s visit	€109	Visit	6	€653	€163	Gamma	[31]
	*Total*				*€9279*			
3×AC/3×DC	Doxorubicin	€204	90 mg	3,2	€653	€163	Gamma	[31]
	Cyclophosphamide	€45	1080 mg	2,7	€120	€30	Gamma	[31]
	Peg-filgrastim	€849	1 mg	3	€2548	€637	Gamma	[56]
	Docetaxel	€959	108 mg	3,3	€3195	€799	Gamma	[31]
	Capecitabine	€27	4500 mg	29,9	€821	€205	Gamma	[31]
	Pharmacy preparation	€45	Per course		€267	€67	Gamma	NKI
	Day care	€286	Day	6	€1718	€430	Gamma	[30]
	Oncologist’s visit	€109	Visit	6	€653	€163	Gamma	[31]
	*Total*				*€9974*			
Monitoring								
MRI scan								
Hospital costs		€163	Scan	1	€163	€41	Gamma	
Specialists fees		€52	Scan	1	€52	€13	Gamma	
*Total*					*€215*			
Confirm incidental findings		€149	Episode	1	€149	€37	Gamma	
Chemotherapy related toxicities								
Neutropenia		€14397	Episode	1	€14397	€425	Gamma	[35]
Vomiting		€92	Episode	1	€92	€23	Gamma	[57]
CHF		€18225	Episode	1	€18225	€4556	Gamma	[33]
MDS/MLA		€112946	Episode	1	€112946	€28236	Gamma	[58, 59]
Health states								
DFS	In & out –patient	€2793	Episode	1	€2793	€563	Gamma	[36]
	Drugs	€79	Episode	1	€79	€20	Gamma	[36]
	*Total*				*€2872*			
R	Local relapse							
	In & out -patient	€12497	Episode	1	€12497	€1692	Gamma	[36]
	Drugs	€2336	Episode	1	€2336	€584	Gamma	[36]
	Distant metastasis							
	In & out -patient	€11645	Episode	1	€11645	€1346	Gamma	[36]
	Drugs	€5772	Episode	1	€5772	€1443	Gamma	[36]
	*Total*				*€16125*			
BC death		€8296	Episode	1	€8296	€2074	Gamma	[36]

*Abbreviations: SE* standard error, *AC* cyclophosphamide, doxorubicine; *DC* docetaxel, capecitabine; *HFS* hand-food-syndrome, *CFH* congestive heart failure, *AML/ADM* acute myeloid leukaemia/myelodysplastic syndrome, *MRI* magnetic resonance imaging, *tp* transition probability, *HR* hazard ratio, *RG-NACT* response guided neoadjuvant chemotherapy, *NACT* neoadjuvant chemotherapy, *DFS* disease free survival, *R* relapse, *RFS* relapse free survival, *BCSS* breast cancer specific survival, *BC* breast cancer, *ATS* acute transition symptom, *NKI* Netherlands Cancer Institute

^a^Dirichlet distribution: mean/SE, Beta distribution: α/β, Normal distribution: mean/SE

^b^We derived these proportions with the dataset of Rigter et al., as explained in the section ‘clinical input parameters’ and following the definitions of ‘Table 2’

^c^We assumed a SE = 0.1

^d^We assumed a SE = 0.01

^e^We assumed SE = 0.25 when this was not available from literature

#### Clinical

The proportions of favourable and unfavourable patients at monitoring and after 5-years of NACT were retrieved from an updated version of the individual patient data from Rigter et al. [10]. The transition probabilities (tp) simulating a relapse and a breast cancer death event were derived from Kaplan-Meyer (KM) curves. The first from a KM of RFS (interval from finishing the NACT intervention to occurrence of first relapse) and the second, from a KM of breast cancer specific survival (BCSS; interval from relapse to occurrence of breast cancer death). The KMs were either constructed uniquely with raw data of Rigter et al. [10], or by using additional assumptions, which we explain in detail below. Calculations were performed in SPSS (IBM Corp. Released 2013. IBM SPSS Statistics for Windows, Version 22.0).

*RG-NACT*: The tps for the group of false-unfavourable and false-favourable patients were derived by using KMs and the formula *tp*(*t*_*u*_) = 1 − *exp*{*H*(*t* − *u*) − *H*(*t*)} [26], where *u* is the length of the Markov cycle (1 year) and *H* is the cumulative hazard. Data for the KM of RFS came from 25 relapsed patients from Rigter et al. [10]*,* and that of BCSS, from literature [27]. The tps of relapse and breast cancer death for the true-favourable and true-unfavourable patients were assumed to be zero at all times, as these patients do not relapse nor die from breast cancer (see Table 2). *Conventional-NACT*: tps were derived from KM curves, with data from the complete dataset of Rigter et al. [10] for the RFS curve and data from literature [27] for the BCSS curve. The formula to derive tps was: *tp*(*tu*) = 1 − *exp*{1/*τ*(*H*(*t* − *u*) − *H*(*t*))} [26], where $\tau$ is the treatment effect or hazard ratio (HR) of RG-NACT vs. conventional-NACT. This formula allowed calculating the tps from a “hypothetical” control arm, which was inexistent in the Rigter et al. [10] study. The used HRs were 0.5 for the RFS curve, and 0.6 for the BCSS curve. Both HRs were derived from literature. They were set equal to the reported HR of DFS and OS in a similar population of ER-positive breast cancers where RG-NACT vs. conventional-NACT was being compared [11]. As these assumptions could affect our cost-effectiveness results, we performed a one-way and two-way sensitivity analysis (SA) to the HRs (range 0.1 - 1.5).

The tps of non-BC related deaths (i.e., transition from any state to D) were accounted for by using Dutch life tables [28]. The occurrence of vomiting, neutropenia, HFS and desquamation under 3×AC and 3×DC, were derived from literature [24]. When a patient received both 3×AC and 3xDC the probability of vomiting and neutropenia was represented as the combined probability of two independent events (P(A and B) = P(A) * P(B)). The probability of occurrence of CHF due to the administration of anthracyclines was accounted for in the 1st-year of the model and was dose-dependent: 0.2 % with 3×AC and 1.7 % with 6xAC [23]. Also the probability of incidental findings at MRI was accounted for in that year [29]. The frequency of MDS and AML events was based on cumulative doses of anthracycline and cyclophosphamide [25]. Patients whose NACT was interrupted to treat toxicities were still assumed to benefit from NACT and the same relapse rate was applied.

#### Costs

*Intervention costs* comprise of chemotherapy, monitoring, chemotherapy-related toxicities and costs of confirming incidental findings. To calculate drug dosages we assumed patients of 60Kg and body-surface area of 1.6 m2. Drug use was derived from study protocol, and costed by using literature [30, 31] and Dutch sources on costs and prices (Dutch National Health Care Institute; Dutch Healthcare Authority; Dutch Health Care Insurance Board). Chemotherapy costs included day care and one visit to the oncologist per cycle. Costs of monitoring consisted of one MRI scan [32] and one medical visit of 1 h (accounting for waiting time) [31]. Costs of treating toxicities were taken from literature [33–35]. Costs of confirming incidental findings were estimated as an average of “standard diagnostic imaging” (i.e., Ultrasound, x-Ray and bone scintigraphy) using prices from the ‘The Nederlandse Zorgautoriteit’ (Dutch Healthcare Authority) as a proxy [32]. Health state costs, i.e., follow up costs for the DFS health state and detection plus treatment costs for the R health state, were derived from literature [36]. All results were reported in 2013 Euros, using exchange currencies [37] and the consumer price index to account for inflation [38].

#### Health-Related Quality of life

Utilities were derived from published literature. The DFS utility was 0.78 except in the 1st-year cycle when patients either accrued the utility of the NACT regimen without toxicities i.e., 0.62 [39], the utility of the NACT regimen with toxicities i.e., 0.62 minus the utility decrements [40–42]) or the utility of anxiety in patients were incidental findings at MRI occurred i.e., 0.68 [43]. These utilities lasted for the whole cycle. The R utility was calculated as an average of the utility of local and distant relapse [39]. All utility weights were obtained from sources using the EuroQoL EQ-5D questionnaires, except anxiety, which was derived from a Quality of Well-Being index [43]. There is no literature to suggest an effect of monitoring on HRQoL, thus this was assumed unaltered.

#### Scenarios and resource modelling

Additional parameters to simulate the scenarios and to perform the resource modelling exercise were added in the model. These include a parameter reflecting the RG-NACT uptake, and parameters illustrating the proportion of i) patients with MRI contraindications (impaired renal function due to the risk of developing Nephrogenic Systemic Fibrosis (NSF) [44], presence of ferrous body parts like peacemaker (mean of values reported in [45–47], and claustrophobia [48]), ii) patients with NSF [49], iii) patients with malignant incidental findings [30] and iv) MRI technologists with acute transition symptoms (ATS) [50].

### Cost-effectiveness analysis

The 5-year cumulative outcomes (health benefits and costs) were simulated for a cohort of 6306 individuals. The cost-effectiveness outcome measure was the incremental cost-effectiveness ratio (ICER), which is the difference in expected costs (per patient) divided by the difference in expected effects expressed as (quality-adjusted) life-years ((QA)LYs)) of treating one hypothetical cohort with RG-NACT vs. treating an identical cohort with conventional-NACT. For the current implementation scenario, we compared the expected costs and QALYs of a cohort as treated with conventional-NACT, to the costs and QALYs of a cohort partially treated with RG-NACT, as dictated by the implementation rate and MRI contraindications. Patients where RG-NACT was not implemented or MRI was contraindicated were modelled as receivers of conventional-NACT. The full implementation scenario was modelled in the same way, except that the RG-NACT strategy was now applied to all patients in the cohort, except those with MRI contraindications receiving conventional-NACT.

We performed a probabilistic sensitivity analysis (PSA) after assigning a distribution to each model parameter following the recommendations by Briggs et al. [38]. A beta distribution was assigned to binomial data such as toxicities and transition probabilities, a dirichlet distribution to the proportions of true/false favourable/unfavourable patients, and a gamma distribution to utilities and costs (Table 3). The uncertainty surrounding the model results was presented as cost-effectiveness acceptability curves (CEAC), which reflect the probability of each alternative to be cost-effective across a range of threshold values for cost-effectiveness. We discounted future costs and health effects at a 4 % and 1.5 % yearly rate respectively, according to the Dutch guidelines on health-economics evaluations [51]. A strategy was considered cost-effective if the ICER did not exceed the willingness-to-pay threshold of €20.000/QALY.

### Resource modelling analysis

We estimated the health services required and the health outcomes experienced in each strategy. Health services required included: number of 1) MRI scans performed, 2) patients scanned per MRI, 3) Full-time equivalent (FTE) MRI technologists, 4) FTE breast radiologists and 5) confirmation of incidental findings. Health outcomes included: number of 1) relapses prevented, 2) breast cancer deaths prevented, 3) excluded patients due to contraindications, 4) patients with adverse events (including NSF, CHF and AML/ADS), 5) patients with anxiety due to incidental findings, 6) patients with malignant incidental findings, and 7) fte MRI technologists with ATS. These outcomes were analysed deterministically for the current and full implementation scenarios and expressed for the 6306 ER-positive/HER2-negative breast cancer women. A detailed description of the calculations and sources for each outcome is presented in (Table 4).

**Table 4:** Resource modelling outcomes, sources and calculations

	Current implementation (16 hospitals, 31 MRIs)	Full implementation (113 hospitals, 148 MRIs)	Source
Health services required at the country level			
No of MRIs scans performed	Calculations in Table 2	No of stage II-III, ER-positive/HER2-negative breast cancers in the Netherlands	See Table 2
No of patients scanned per MRI	‘No of MRI scans performed’/31 MRIs^a^	‘No of MRI scans performed’/148 MRIs^a^	See footnote a
Fte MRI technologists required	Yearly hours required of MRI technologist to perform the ‘No of MRIs scans performed’/Fully workable hours of an MRI technologist a year^b^	idem	See footnote b
Fte breast radiologists required	Yearly hours required of breast radiologist to perform the ‘No of MRIs scans performed’/Fully workable hours of a breast radiologist a year^c^	idem	See footnote c
No of confirmations of incidental findings (using standard imaging)	Derived from the Markov model	idem	-
Health services required at the hospital level			
No of MRIs scans performed per hospital	‘No of MRI scans performed’/16 hospitals^d^	‘No of MRI scans performed’/113 hospitals^e^	See footnote d and e
No of patients scanned per MRI per hospital	‘No of MRI scans performed per hospital’/mean MRIs per hospital^a^	‘No of MRI scans performed per hospital’/mean MRIs per hospital^a^	See footnote a
Fte MRI technologists required per hospital	Yearly hours required of MRI technologist to perform the ‘No of MRI scans performed per hospital’/Fully workable hours of an MRI technologist a year^b^	idem	See footnote b
Fte breast radiologists required per hospital	Yearly hours required of breast radiologist to perform the ‘No of MRI scans performed per hospital’/Fully workable hours of a breast radiologist a year^c^	idem	See footnote c
Health outcomes gained at the country level			
No of relapses prevented	Derived from the Markov model	idem	-
No of breast cancer deaths prevented	Derived from the Markov model	idem	-
Health outcomes lost at the country level			
No of excluded patients due to contraindications	Derived from the Markov model	idem	-
No of patients with NFS	‘No of MRI scans performed’* *p* of NSF	idem	[48]
Fte MRI technologists with ATS	‘Fte MRI technologists required’* *p* of ATS	idem	[49]
No of patients with CHF	Derived from the Markov model	idem	-
No of patients with long term AML/ADS	Derived from the Markov model	idem	-
No of patients with anxiety due to incidental findings	Derived from the Markov model	idem	-
No of patients with malignant incidental findings	‘No of confirmations of incidental findings’ *p malignant incidental findings^f^	idem	[28]

*Abbreviations: No* number, *Fte* full-time equivalent, *MRI* magnetic resonance imaging, *RG-NACT* response guided neoadjuvant chemotherapy; *p* probability, *NSF* nephrogenic systemic fibrosis, *ATS* acute transient symptom, *CHF* chronic heart failure, *DSF* disease free survival, *R* relapse, *AML/ADS* myelodysplastic syndrome/acute myeloid leukaemia

Note that when a calculation refers to another outcome of the table this is always the outcome within the same column i.e., within the same implementation rate

Idem means calculated equal as the left cell, but adapted to the full implementation scenario figures

^a^We search for this information in each hospital website. When this information was not available or unclear, we made use of literature [49] where the most frequent quantity of MRIs per type of hospital is presented (three for academic hospitals and one for general hospitals)

^b^Hours required of MRI technologists for the ‘No of MRIs scans performed (per hospital)’ in a year are calculated by assuming that a full scanning procedure requires 1 h of MRI technologist. Employees were assumed to work 52 weeks/year, 5 days/week i.e., 260 days/year. Of these, 40 days would be vacation and sick days, resulting thus in 220 workable days/year. Assuming workers are employed for 8 h/day this results in 1760 working hours/year. Yet workers need some time off during their working days i.e., breaks, assumed to be 20 %. Thereby, a fully workable year is of 1408 h

^c^Hours required of breast radiologist for the ‘No of MRIs scans performed (per hospital)’ in a year are calculated by assuming a mean of 6.8 min needed for a breast radiologist to interpret one MRI scan [53]. The workable hours a year of a breast radiologist were calculated exactly as explained in footnote 2

^d^Assuming its use in the biggest Dutch hospital network involved in RG-NACT (see ‘resource modelling analysis’ section)

^e^Assuming its use in all Dutch hospitals (locations) with MRI expected to deliver cancer treatment (i.e., university, general and specialized hospitals) (see ‘resource modelling analysis’ section)

^f^After confirming by ultrasound

Volumes of health services needed were also calculated at the hospital level, which required determining the number of hospitals expected to offer RG-NACT under each scenario. For current implementation, we assumed RG-NACT to be used in the 16 hospitals of the largest Dutch hospital network currently involved in the RG-NACT trial NCT01057069 (Clinical Trials.gov). Although this trial excludes ER+ patients, we expected involved hospitals to have endorsed RG-NACT in other subtypes with single institution studies, as is the case in the NKI. For the full implementation, we considered all 113 hospitals (locations) with MRI that deliver cancer treatment (i.e., university, general and specialized hospitals), as identified from the database published by the National Public Health Atlas [52]. The presence and quantity of MRI scans per hospital was either taken from that hospital’s website or based on literature [50], indicating 3 MRIs per academic hospital and 1 per general hospital.

As increasing RG-NACT uptake from 4 to 100 % is not realistic in a short time-frame, we explored the resource requirements and health outcomes across a range of implementation rates via one-way SA including 20, 40, 60 and 80 % uptake.

All assumptions made were confirmed by an experienced MRI technologist in a general hospital. One-way SAs on one key-assumptions was done: ‘the time required by a breast radiologist for MRI scan interpretation’ (range 6.8–15 min).

## Results

### Cost-effectiveness analysis

**Fig. 2:**
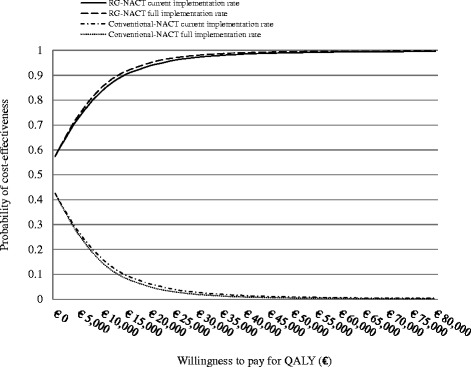
Cost effectiveness acceptability curves. At a willingness to pay threshold of €20.000/QALY, RG-NACT is expected to be the optimal strategy with 94 and 95 % certainty under the current and full implementation scenarios respectively

At current implementation (4 %) RG-NACT was expected to result in 0.005 QALYs gains and savings of €13 per patient. Under full implementation, RG-NACT is expected to generate 0.12 additional QALYs and savings of €328 per patient (Table 5). In both scenarios, RG-NACT is expected to dominate (be more effective and less costly) than conventional-NACT. The results of the PSAs show that at a willingness to pay threshold of €20.000/QALY, RG-NACT is expected to be the optimal strategy under the current and full implementation scenarios, with 94 and 95 % certainty respectively (Fig. 2).

**Table 5:** Resource modelling and cost-effectiveness results for the current and full implementation scenarios of response-guided NACT in the Netherlands

Cost-effectiveness analysis expressed per patient												
	Current implementation (4 %)						Full implementation (100 %)					
	Costs (€)	LYs	QALYs	∆ costs (€)	∆ QALYs	ICER	Costs (€)	LYs	QALYs	∆ costs (€)	∆ QALYs	ICER
RG-NACT disc	28013	4.58	3.46	−13	0.005	dominant^a^	27698	4.64	3.58	−328	0.12	dominant
RG-NACT undisc	30362	4.79	3.62	−14	0.005	dominant	30021	4.85	3.74	−355	0.13	dominant
Conventional-NACT disc	28026	4.58	3.45	-	-	-	28026	4.58	3.45	-	-	-
Conventional-NACT undisc	30377	4.76	3.61	-	-	-	30377	4.76	3.61	-	-	-
One-way and two-way sensitivity analysis												
	ICER				ICER					ICER		
HR RFS			HR OS				HR RFS/BCSS					
0.1	€-12857/QALY (cost-effective)		0.1		€1190/QALY (cost-effective)		0.1/0.1			€-922/QALY (cost-effective)		
1	€2398/QALY (cost-effective)		1		€-10692/QALY (cost-effective)		1/1			€1139/QALY (cost-effective)		
1.5	€9367/QALY (cost-effective)		1.5		€-15507/QALY (cost-effective)		1.5/1.5			€10299/QALY (cost-effective)		
Resource modelling analysis expressed in relation to the Dutch population of ER-positive/HER2-negative breast cancer women (*n* = 6306)^c^												
					Current implementation (16 hospitals, 31 MRIs)		Full implementation (113 hospitals, 148 MRIs)			Transition from current to full implementation		
Health services required at the country level												
No of MRIs scans performed					218		5335			+5117		
No of patients scanned per MRI					7		36			+29		
Fte MRI technologists					0.2		3.8			+3.6		
Fte breast radiologists					0.02		0.4			+0.4		
					0.04^b^ (↑121 %)		0.95^b^ (↑121 %)					
No of confirmations of incidental findings (using standard imaging)					38		939			+901		
Health services required at the hospital level												
No of MRIs scans performed per hospital					14		47			+33		
No of patients scanned per MRI per hospital					7		36			+29		
Fte MRI technologists per hospital					0.01		0.03			+0.02		
Fte breast radiologists per hospital					0.001		0.004			+0.003		
					0.002^b^ (↑121 %)		0.001^b^ (↑121 %)					
Health outcomes gained at the country level												
No of relapses prevented					0.4		9			+9		
No of breast cancer deaths prevented					6		149			+143		
Health outcomes lost at the country level												
No of excluded patients due to contraindications					40		971			+931		
No of patients with NFS					0.07		2			+2		
Fte MRI technologists with acute transient symptom					0.04		0.9			+1		
No of patients with CHF					106		83			−23		
No of patients with long term AML/ADS					23		21			−2		
No of patients with anxiety due to incidental findings					38		939			+901		
No of patients with malignant incidental findings					8		192			+184		

*Abbreviations: Disc* discounted, *undisc* undiscounted, *No* number, *Fte* full-time equivalent, *MRI* magnetic resonance imaging, *NSF* nephrogenic systemic fibrosis, *ATS* acute transient symptom, *CHF* chronic heart failure, *AML/ADS* myelodysplastic syndrome/acute myeloid leukaemia

^a^RG-NACT is more effective and less costly than conventional NACT

^b^if radiologists spent 15 min to interpret 1 MRI scan

^c^When possible, figures were rounded to the nearest whole number

SAs of RFS and BCSS hazard ratios (baseline values of 0.5 and 0.64 respectively), invariably showed the RG-NACT strategy to be cost-effective (Table 4). Even when LYs were slightly higher in the conventional-NACT arm (i.e., with HRs of >1), the better quality of life provided by the DC treatment of the RG-NACT strategy (lower and better tolerated adverse events) maintained the incremental QALYs for the RG-NACT strategy.

### Resource modelling analysis

Under the current implementation scenario we calculated that over 5-years, the RG-NACT strategy requires 218 MRI scans to be performed in the target population of 6306 women, after 40 exclusions due to contraindications. With 31 MRI scans currently used for this purpose (estimated number of MRI scans in the multicentre NCT01057069 trial), 7 patients were scanned/MRI, requiring a total of 0.2 fte MRI technologists and 0.02 fte breast radiologists. At the hospital level covering a population of 6306 breast cancers, 14 MRI scans would be required for the prevalent population over a 5-year timeframe. Assuming an average capacity of 2 MRI scans/hospital (estimated weighted average of MRI scans/hospital within the multicentre NCT01057069 trial), this would translate to 7 patients scanned/MRI, demanding 0.01 fte MRI technologists and 0.001 fte breast radiologists per hospital. In terms of health outcomes, the current implementation scenario was expected to prevent 0.4 relapses and 6 breast cancer deaths, while yielding 0.07 patients with NSF. Besides, 106 patients would have a CHF, 23 patients would suffer from AML/ADS and 38 incidental findings were expected, of which 8 would be malignant. Of the required 0.2 fte MRI technologists, 0.04 fte would suffer from ATS (Table 4).

Under the full implementation scenario, we calculated that 5335 MRI scans would be needed over a 5-year period for the 6306 pertinent breast cancer population, after excluding 971 patients for contraindications. With 148 MRI scans available (estimated number of MRI scans in the estimated 113 hospitals), this would require 36 patients to be scanned/MRI for which 3.8 fte MRI technologists and 0.4 fte radiologists are needed. At the hospital level, 47 MRI scans are expected to be performed for the prevalent population of 6306 within 5-years. Assuming the mean MRI scans/hospital is 1.3 (estimated weighted average of MRIs/hospital within the estimated 113 hospitals), 36 patients would be scanned per MRI, requiring 0.03 fte MRI technologists and 0.004 fte breast radiologists per hospital. In terms of health outcomes, the full implementation scenario was expected to prevent 9 relapses and 149 breast cancer deaths, but to bring about 2 patients with NSF, 83 patients with CHF and 21 patients with AML/ADS. Furthermore, there are 939 incidental findings expected, of which 192 would be malignant, and 0.9 fte MRI technologists are projected to get ATS (Table 4).

The transition from current (4 %) to full (100 %) implementation is expected to increase the number of examinations by 5117 (2347 %) countrywide or by 33 (247 %) per hospital, consequently demanding an increase of scan utilization (for an additional 29 patients), an increase in the number MRI technologists by 3.6 fte countrywide or by 0.02 fte per hospital, and a marginal increase in breast radiologists by 0.4 fte countrywide or by 0.003 fte per hospital. In terms of health outcomes, full implementation would diminish the number of breast cancer related deaths and relapses by 25-fold (from 6 to 149) and 23-fold (from 0.4 to 9) respectively, and the number of CHF and AML/MDS by ~0.8-fold (from 106 to 83) and ~0.9-fold (from 23 to 21) respectively. However, these would come at the cost of a ~25-fold increase on health losses (additional 2 patients with NSF, 1 fte MRI technologist with ATS, 901 patients with anxiety due to presence of incidental findings, and 184 patients with confirmed malignant findings).

**Fig. 3:**
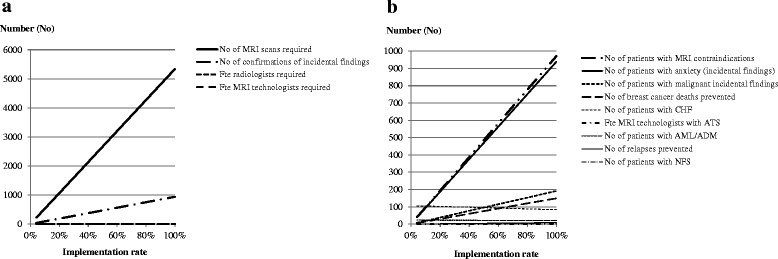
Influence of implementation rates on resource modelling outcomes, (**a**) on health services required and (**b**) on health outcomes. Abbreviations: No = number; Fte = full-time equivalent; MRI = magnetic resonance imaging; ATS = acute transition syndrome; CHF = chronic heart failure; AML/ADM = acute myeloid leukaemia/myelodysplastic syndrome; NFS = nephrogenic systemic fibrosis

The one-way SA to the RG-NACT uptake rate showed that increasing rates markedly increases the number of patients with MRI contraindications, the number confirmatory scans and the number of patients with anxiety while awaiting for those (Fig. 3). Simultaneously, the number of cancer deaths, and the number of patients with CHF and AML/ADS decreased consistently (by ~1.5, ~0.98 and ~0.95 -fold per 20 % rate increase).

The results of the one-way SA on the radiologists’ working pattern assumption showed that increasing the time required for MRI scan interpretation to 15 min, increased the ‘fte breast radiologists’ required by 121 % (Table 4).

## Discussion

The aim of our study was to estimate the cost-effectiveness and resource requirements of implementing RG-NACT with MRI for ER-positive/HER2-negative breast cancer patients using The Netherlands as a case study population. As RG-NACT is an emerging treatment approach and its implementation is at its onset, we performed these analyses under a current implementation scenario of 4 % uptake, and under a full implementation scenario, to anticipate the outcomes of a potential wider roll-out.

At the current 4 % uptake RG-NACT is expected to be less expensive and achieve more QALYs than conventional-NACT. With higher implementation rates, more patients will be treated with this cost-saving and effective strategy, rendering RG-NACT ever more dominant. At full implementation, 0.12 additional QALYs and savings of €328 per patient are expected. This is achieved despite 15 % (971 out of the 6303 patients) being treated with conventional-NACT due to MRI contraindications. In both scenarios, decision uncertainty surrounding the ICERs is low (~5 %).

The main drivers of advantageous survival in the RG-NACT are the HRs used to derive the hypothetical survival of the conventional-NACT strategy. Either of the HRs used (for RFS and BCSS) was below 1, thus implying less breast cancer related events in the RG-NACT strategy compared to the conventional-NACT strategy. These values were based on best available data from the GeparTrio trial [11], but this evidence is still preliminary. One- and two-way SA of these HR values demonstrated that even when survival was higher in the conventional-NACT strategy, the better quality-of-life derived from DC treatment in the RG-NACT strategy maintained the cost-effectiveness of RG-NACT.

The cost savings of RG-NACT hinge on a satisfactory diagnostic performance of MRI. Under current diagnostic performance, 79 % of patients would not yield any event in the RG-NACT strategy, compared to 76 % in conventional-NACT. Although the prevention of these events came at the costs of 30 % of patients receiving a more expensive treatment than conventional-NACT (>€695), as treating one relapse is even more expensive (€16125), RG-NACT was still cost saving.

The resource modelling analysis showed that increasing RG-NACT uptake rates from 4 to 100 % is expected to increase the number of examinations by 5117 (2347 %), consequently demanding a 5-fold increase in scans utilization, a 19-fold increase in the number MRI technologists and a 20-fold increase in the number of breast radiologists. Thereby, adapting current practice to meet these resources requires paying special attention to the availability and utilization of MRIs, as well as availability of technical personnel. For instance, fully implementing RG-NACT in the Netherlands, where 5701 MRI examinations were performed in 2013 (considering 843765 MRI examinations [15] performed in 148 MRIs), would only require 2 days of additional MRI scanning per year. However, current MRI utilization is already intense; considering 1 scan lasts 1/2 h and the scan works 8 h/day, 843765 MRI examinations results in 356 days of MRI scanning. As there are only 260 workable days a year, hospitals had to intensify MRI’s use i.e., by adding extra evening shifts. Hence, adding 2 extra days of scanning a year would require of an even more intense MRI utilization. In terms of personnel, the number of required MRI technologists and breast radiologists are not expected to be a limiting implementation factor. While fully implementing RG-NACT would require additional 2 fte MRI technologists and 1 fte breast radiologists to the current 403 fte MRI technologists and 91 fte breast radiologists required a year, availability is estimated to be of 1700 MRI technologists countrywide [50] and 10 breast radiologists per hospital [53].

In terms of health outcomes gained, full implementation would diminish the number of breast cancer related deaths and relapses by 25- and 23-fold respectively, and the number of severe and costly adverse events as CHF and AML/MDS by ~0.8- and ~0.9-fold respectively. However, these would come at the cost of a parallel ~25-fold increase in patients with NSF, MRI contraindications, MRI technologists with ATS and incidental findings causing anxiety and other diseases.

Our post-hoc analysis on resource requirements at various RG-NACT implementation rates allow identifying those that seem feasible given current resources. Considering current MRI machines and personnel capacity, RG-NACT implementation seems feasible at any rate. However, it would be interesting to further investigate whether there is sufficient capacity to handle an increase of incidental findings (requiring further diagnostic examinations), as well the cost-consequences of treating those that are diagnosed as malignant.

Our study has some limitations. A limitation of the response-guided approach itself was the impossibility to distinguish in the false-unfavourable group, patients truly falsely classified at monitoring from patients irresponsive to 3×DC or NACT in general. Yet, as this is inherent to guided-NACT, it was included as such in the model. Furthermore, we did not consider adjuvant treatment in our model, as the administration of this was similar between arms. Moreover, we considered AC, instead of a 3rd generation regimen containing taxanes as standard treatment because it was considered the best comparator for the used RG-NACT regimens. As costs of those are different, we performed a post-hoc one-way SA and found that RG-NACT would become more dominant due to increased cost savings. Additionally, we only accounted for direct-medical costs as other cost beyond the direct hospital-based treatment, such as productivity losses or home health care exist, are less likely to influence decision-making.

## Conclusion

While the typical CEA assumes perfect implementation of the strategy under investigation, we showed the impact of implementation rates on incremental health gains and cost-savings of RG-NACT in the Dutch population of ER-positive/HER2-negative breast cancers. Furthermore, we showed that fully implementing RG-NACT generates a ~24-fold increase in health benefits, but requires MRI and personnel capacity to be increased by 5- and ~20-fold. In the Netherlands, personnel capacity is likely to be sufficient for a full implementation scenario, but MRI utilization needs to be intensified.

## Abbreviations

AC, doxorubicin and cyclophosphamide; AML, acute myeloid leukaemia; ATS, acute transition symptoms; BCSS, breast cancer specific survival; CEA, cost effectiveness analysis; CEAC, cost-effectiveness acceptability curves; CHEERS, Consolidated Health Economic Evaluation Reporting Standards; CHF, congestive heart failure; D, death; DC, docetaxel and capecitabine; DFS, disease free survival; ER, oestrogen receptor; Fte, full time equivalent; HER2, human epidermal growth factor receptor-2; HFS, hand food syndrome; HR, hazard ratio; HRQoL, health related quality of life; ICER, incremental cost-effectiveness ratio; KM, Kaplan Meyer; LY, life years; MDS, myelodysplastic syndrome; MRI, magnetic resonance imaging; NACT, neoadjuvant chemotherapy; NFS, nephrogenic systemic fibrosis; NKI, Netherlands Cancer Institute; pCR, Pathologic complete response; PSA, Probabilistic sensitivity analysis; QALYs Quality-adjusted-life-years; R, Relapse; RFS, Relapse free survival; RG-NACT, Response-guided neoadjuvant chemotherapy; SA, Sensitivity analysis; Tp, Transition probabilities
